# Cytidine Alleviates Dyslipidemia and Modulates the Gut Microbiota Composition in ob/ob Mice

**DOI:** 10.3390/nu15051147

**Published:** 2023-02-24

**Authors:** Kaixia Niu, Pengpeng Bai, Junyang Zhang, Xinchi Feng, Feng Qiu

**Affiliations:** State Key Laboratory of Component-based Chinese Medicine, School of Chinese Materia Medica, Tianjin University of Traditional Chinese Medicine, Tianjin 301617, China

**Keywords:** cytidine, dyslipidemia, gut microbiota, ob/ob mice

## Abstract

Cytidine and uridine are endogenous metabolites in the pyrimidine metabolism pathway, and cytidine is a substrate that can be metabolized into uridine via cytidine deaminase. Uridine has been widely reported to be effective in regulating lipid metabolism. However, whether cytidine could ameliorate lipid metabolism disorder has not yet been investigated. In this research, ob/ob mice were used, and the effect of cytidine (0.4 mg/mL in drinking water for five weeks) on lipid metabolism disorder was evaluated in terms of an oral glucose tolerance test, serum lipid levels, liver histopathological analysis and gut microbiome analysis. Uridine was used as a positive control. Our findings reveal that cytidine could alleviate certain aspects of dyslipidemia and improve hepatic steatosis via modulating the gut microbiota composition in ob/ob mice, especially increasing the abundance of short-chain fatty acids-producing microbiota. These results suggest that cytidine supplementation could be a potential therapeutic approach for dyslipidemia.

## 1. Introduction

Dyslipidemia is a lipid disorder characterized by an elevation in the level of serum total cholesterol (TC), triglyceride (TG) or low-density lipoprotein cholesterol (LDL-C) and a low level of high-density lipoprotein cholesterol (HDL-C). Various diseases, such as cardiovascular complications, fatty liver, diabetes, obesity and atherosclerosis, are clearly associated with dyslipidemia [1]. Intervention at the early stage of dyslipidemia can prevent the occurrence of associated diseases effectively [2]. Recently, the gut microbiota has been reported to play an important role in hosting lipid metabolism, which is mediated via metabolites produced by gut microbiota, such as short-chain fatty acids, bile acids and lipopolysaccharide [3,4,5].

Nucleotides, the building blocks of RNA and DNA, play important roles in various biological processes, including gastrointestinal function, energy metabolism and immune regulation [6]. Nucleotides are widely present in food, and they are also used as additives for infant formulas due to the beneficial effects provided to infants [7]. Among all the nucleotides, uridine has been extensively investigated and is reported to be able to ameliorate lipid accumulation via modulating gut microbiota composition [8,9,10,11,12]. Our previous study found that a triterpene acid derived from centella, named asiatic acid, could alleviate metabolic disorders in ob/ob mice, and the levels of cytidine in the liver were significantly increased [13]. This prompted the hypothesis that the supplementation of cytidine might alleviate metabolic disorders. Moreover, cytidine is a substrate for the salvage pathway of pyrimidine synthesis and could be metabolized into uridine via cytidine deaminase in vivo [14]. Thus, it was speculated that besides uridine, cytidine treatment might also show an alleviation effect on dyslipidemia. However, until now, the effect of cytidine on lipid metabolism has not been investigated.

The aim of this study was to investigate whether cytidine treatment could alleviate metabolic disorders. Uridine was applied as a positive control, and ob/ob mice were used in this study. Moreover, the effect of cytidine on the gut composition in ob/ob mice was also studied.

## 2. Materials and Methods

### 2.1. Chemicals, Animals and Administration

Cytidine (99% purity) and uridine (99% purity) were purchased from Shanghaiyuanye Bio-Technology Co., Ltd. (Shanghai, China); Ultrapure water was purchased from Watsons Group, and all other reagents used in this study were of analytical grade. Male SPF (specific pathogen-free)-grade ob/ob mice (5 weeks old) were obtained from GemPharmatech Biotechnology Co., Ltd. (Nanjing, China), and male SPF-grade C57BL/6J mice (5 weeks old) were obtained from SPF (Beijing, China) Biotechnology Co., Ltd. (Beijing, China). All animals were kept in a controlled environment with free access to food and water (temperature, 25 ± 1 °C; humidity, 50 ± 5%; 12 h light/12 h dark cycle) and allowed to adapt to their living conditions for one week.

After acclimatization, 24 ob/ob mice aged six weeks were randomly divided into three groups as follows: model group, cytidine group, and uridine group (n = 8 per group). C57BL/6J mice were applied as a control group (n = 8). Mice in the model group and control group were fed with normal drinking water. Mice in the cytidine group and uridine group were fed with drinking water containing cytidine or uridine at a dose of 0.4 mg/mL for 5 consecutive weeks. All mice were fed a standard diet. Body weights were recorded every week, and water and food consumption were measured every day. By the end of the experiment, mice were anesthetized with pelltobarbitalum natricum. Serum samples used for the determination of biochemical indicators were collected before the mice were sacrificed via cervical dislocation. The liver and cecum were manually isolated. In order to conduct the histological examination, liver tissues were preserved in 4% paraformaldehyde. The cecal contents were collected, and five samples for each group were randomly selected for microbiome analysis. All samples were stored at −80 °C.

### 2.2. Oral Glucose Tolerance Test (OGTT)

After the mice were treated with cytidine or uridine for 5 weeks, oral glucose tolerance was tested. Briefly, all mice first fasted for 12 h, and then each mouse was intragastrically administered glucose solution at a dose of 2 g/kg. Blood glucose concentrations were determined using tail–tip blood sampling at 0, 15, 30, 60, 90, and 120 min after glucose was administered, and the area under the curve (AUC) was calculated.

### 2.3. Biochemical Indicator Detection and Histopathological Analysis

The contents of serum TC, TG, HDL-C and LDL-C were determined using commercial assay kits supplied by Nanjing Jiancheng Bioengineering Institute (Nanjing, China).

Liver tissues were fixed in 4% paraformaldehyde, dehydrated, blocked with paraffin, and then sectioned and stained with hematoxylin and eosin (H&E). Histopathological changes were analyzed and recorded using a light microscope (200×) (OLYMPUS CKX43, OLYMPUS, Tokyo, Japan). The hepatic fatty vacuole area ratio in liver tissue slices was determined by quantifying the amount of stained area using Image J software (version 1.48r, National Institutes of Health, Bethesda, MD, USA).

### 2.4. Gut Microbiome Analysis

Regarding gut microbiome analysis, bacterial DNA from the cecal contents was extracted, and the DNA concentration was detected using 1% agarose gel electrophoresis. DNA encoding the 16S rRNA V3–V4 region was amplified using a pair of universal primers (338F and 806R). The PCR instrument was ABI GeneAmp^®^9700 (ABI, Foster City, CA, USA), and the following reaction conditions were used: 95 °C for 3 min, 27 cycles at 95 °C for 30 s, 55 °C for 30 s, 72 °C for 45 s and finally 72 °C for 10 min.

The sequencing and data analysis were performed based on the quality control software fastp (https://github.com/OpenGene/fastp, accessed on 22 February 2023, v0.19.6) and FLASH (https://ccb.jhu.edu/software/FLASH/index.shtml, accessed on 22 February 2023, v1.2.11). Chimeras were removed, and operational taxonomic unit (OTU) clustering was obtained according to 97% similarity using the UPARSE software platform (http://www.drive5.com/uparse/, accessed on 22 February 2023, v7.0.1090). The RDP classifier (https://sourceforge.net/projects/rdp-classifier/, accessed on 22 February 2023, v2.11) was applied for the acquisition of species classification information for each OTU, and the database SILVA (https://www.arb-silva.de/, accessed on 22 February 2023, v138) was utilized to classify OTUs taxonomically. Bioinformatics, including α-diversity and β-diversity analyses, were subsequently conducted. The Kruskal–Wallis rank sum test was conducted for statistical difference analysis among groups (the significance level was 0.95). The false discovery rate (FDR) approach was applied for multiple comparisons. LEfSe analysis was performed to detect features differentially represented between different groups. The different species in each group were ranked by effect size after linear discriminant analysis (LDA).

### 2.5. Statistical Analysis

Statistical analysis was conducted with the GraphPad Prism 9 software (GraphPad Software, San Diego, CA, USA) followed by one-way analysis of variance (ANOVA) or the Kruskal–Wallis rank sum test. All data are expressed as the mean ± standard deviation. A *p*-value less than 0.05 was considered statistically significant.

## 3. Results

### 3.1. Effect of Cytidine on Body Weight, Water and Food Intake, and Serum Lipid Level

**Figure 1:**
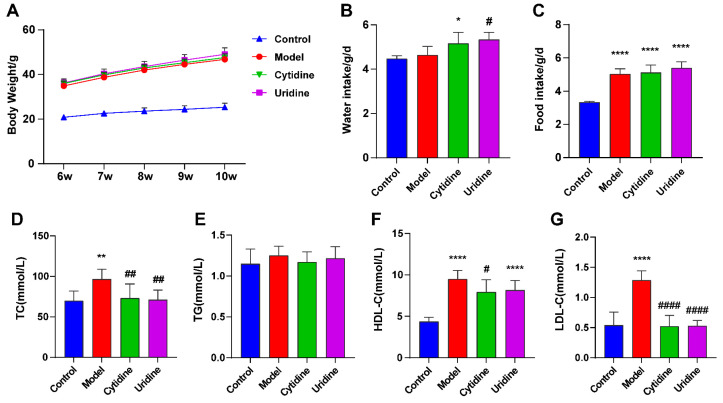
Effect of cytidine on body weight (**A**), water intake (**B**), food intake (**C**), serum total cholesterol (TC) (**D**), total triglyceride (TG) (**E**), high-density lipoprotein cholesterol (HDL-C) and (**F**) low-density lipoprotein cholesterol (LDL-C) (**G**) levels in ob/ob mice. Data are expressed as mean ± SD (n = 8). * *p* < 0.05, ** *p* < 0.01, **** *p* < 0.0001 compared with control group; # *p* < 0.05, ## *p* < 0.01, #### *p* < 0.0001 compared with model group.

As shown in Figure 1, the body weight and food intake of ob/ob mice were significantly higher than those of C57BL/6J mice (*p* < 0.001). Cytidine and uridine treatment had no effect on the body weight and food intake of ob/ob mice, indicating that cytidine and uridine had no obvious toxic effects. Compared with the model group, uridine treatment significantly increased the water intake (*p* < 0.05). Even though no significant difference in water intake was observed between the model group and the cytidine group, an increasing trend was observed after cytidine treatment. Regarding the serum lipid levels, compared with C57BL/6J mice, ob/ob mice showed dyslipidemia symptoms with significantly higher levels of serum TC, HDL-C and LDL-C (*p* < 0.01, *p* < 0.001 and *p* < 0.001, respectively). After five weeks of treatment, cytidine and uridine significantly reduced the levels of TC and LDL-C (*p* < 0.01 and *p* < 0.001, respectively). Cytidine treatment significantly reduced the serum HDL-C level (*p* < 0.05). Regarding the level of HDL-C, a decreasing trend was observed in the uridine group when compared with the model group; however, there was no significant difference.

### 3.2. Effect of Cytidine on OGTT, Liver Index, and Hepatic Steatosis

**Figure 2:**
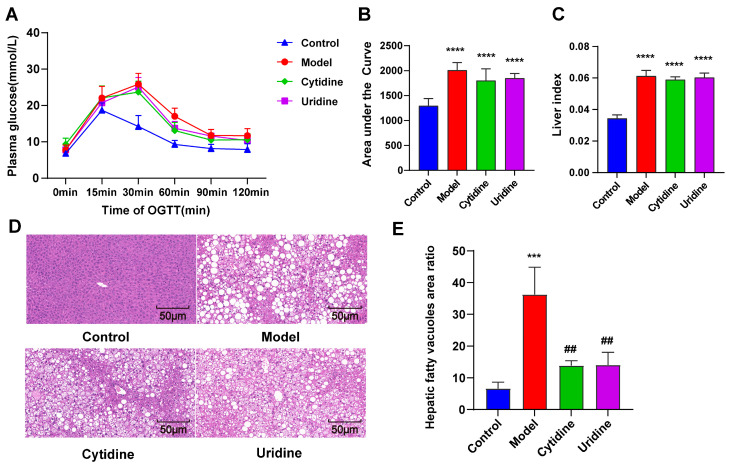
Effect of cytidine on oral glucose tolerance test (OGTT) (**A**,**B**), liver index (**C**), hepatic steatosis (**D**) and hepatic fatty vacuoles area ratio (**E**) in ob/ob mice. Data are expressed as mean ± SD (n = 8). *** *p* < 0.001, **** *p* < 0.0001 compared with control group; ## *p* < 0.01 compared with model group.

In order to investigate whether cytidine could alleviate glucose intolerance in ob/ob mice, OGTT was conducted. As shown in Figure 2, cytidine and uridine treatment could not alleviate the glucose intolerance in ob/ob mice, even though the AUC values of cytidine and uridine groups showed a slight decrease.

The liver index was calculated at the end of the treatment (Figure 2C). A significant increase in the liver index was observed in the model group compared with the control group. However, cytidine and uridine treatment could not alleviate the increase in the liver index in ob/ob mice. Liver tissues were stained with H&E to visualize the histopathological changes in the liver. As shown in Figure 2D,E, obvious hepatic steatosis with greater fat deposition and bigger fat vacuoles was observed in ob/ob mice. After cytidine or uridine treatment, the hepatic steatosis was alleviated. Overall, the above results indicate that cytidine has a beneficial effect that improves hepatic steatosis in ob/ob mice.

### 3.3. Effect of Cytidine on Gut Microbiota Composition in ob/ob Mice

**Figure 3:**
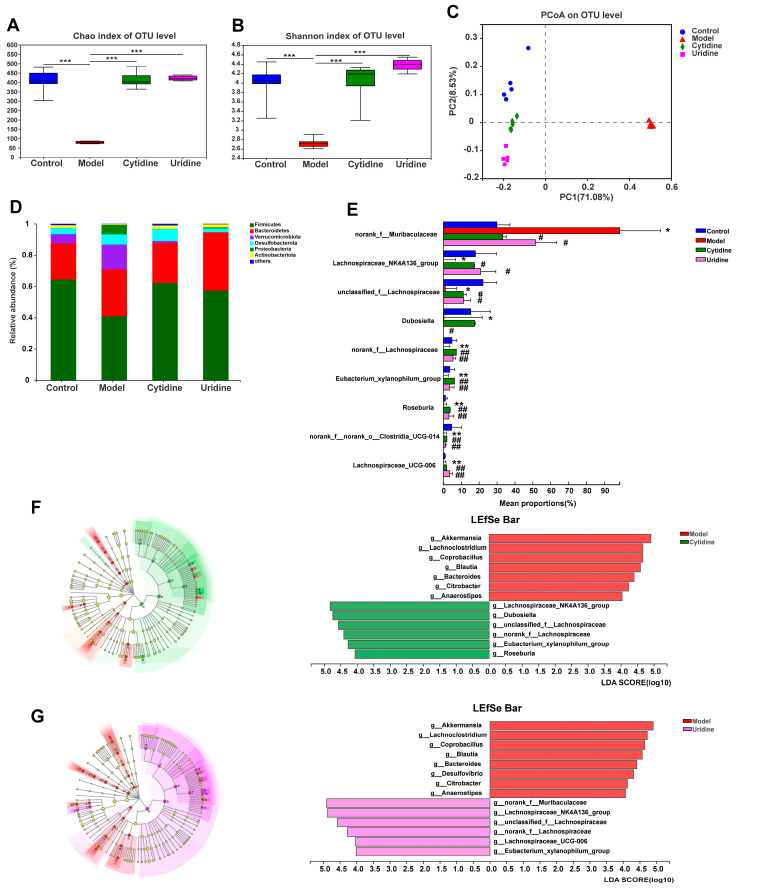
Effect of cytidine and uridine on the structure of gut microbiota in ob/ob mice: (**A**) Chao index, (**B**) Shannon diversity index, (**C**) principal coordinate analysis (PCoA) score plot, (**D**) gut microbiota composition at the phylum level, (**E**) differential abundance of gut microbiota taxa at the genus level, (**F**) linear discriminant analysis (LDA) scores of differentially abundant gut microbiota taxa between the cytidine group and model group using LEfSe method (LDA > 4 and *p* < 0.05) and (**G**) LDA scores of differentially abundant gut microbiota taxa between the uridine group and model group (LDA > 4 and *p* < 0.05). Data are expressed as the mean ± SD (n = 5). * *p* < 0.05, ** *p* < 0.01, *** *p* < 0.001 compared with the control group; # *p* < 0.05, ## *p* < 0.01 compared with model group.

The 16S rRNA sequencing analysis was applied to assess the effects of cytidine and uridine treatment on the composition and abundance of the gut microbiota in the ob/ob mice. Both the Chao and Shannon indexes revealed (Figure 3A,B) that the abundance and diversity of gut microbiota in ob/ob mice were significantly increased after cytidine and uridine treatment (*p* < 0.001). The principal coordinate analysis (PCoA) was then performed on all samples using the unweighted-unifrac distance algorithm to investigate the similarities and differences in bacterial community structures. As shown in Figure 3C, each group of samples showed a fine aggregation state, and the model group was clearly distinguished from the control group. Moreover, the control group, cytidine group and uridine group were at the same latitude. In summary, the composition and abundance of the gut microbiota in ob/ob mice were modulated after cytidine or uridine treatment.

Figure 3D shows the gut microbiota composition at the phylum level, and *Firmicutes* and *Bacteroidetes* were the dominant phyla. After supplementation with dietary cytidine and uridine, the gut microbiota composition returned to normal at the phylum level. At the genus level, when compared with the control group, the abundances of several gut microbiota taxa were significantly decreased in ob/ob mice, including *Lachnospiraceae_NK4A136_group* (*p* < 0.05), *unclassified_f__Lachnospiraceae* (*p* < 0.05), *Dubosiella* (*p* < 0.05), *norank_f__Lachnospiraceae* (*p* < 0.01), *Eubacterium_xylanophilum_group* (*p* < 0.01), *Roseburia* (*p* < 0.01), *norank_f__norank_o__Clostridia_UCG-014* (*p* < 0.01) and *Lachnospiraceae_UCG-006* (*p* < 0.01). Compared with the model group, all of the above-mentioned gut microbiota taxa except for *Dubosiella* were significantly increased after cytidine or uridine treatment (*p* < 0.05 or *p* < 0.01) (Figure 3E). Regarding *Dubosiella*, an obvious increase in the abundance was observed after cytidine treatment; however, there was no statistical difference between the cytidine and model groups. Additionally, when compared with the control group, the abundance of *norank_f__Muribaculaceae* was significantly increased in the model group (*p* < 0.05), and after treatment with cytidine or uridine, the abundance of *norank_f__Muribaculaceae* was significantly decreased compared with the model group (*p* < 0.05) (Figure 3E). 

To identify the differences in gut microbiota composition among different groups, LEfSe was utilized for the screening of differential species (LDA > 4). The results show that when compared with the model group, the differential gut microbiota taxa enriched in the cytidine group included *g__Lachnospiraceae_NK4A136_group*, *g__Dubosiella*, *g__unclassified_f__Lachnospiraceae*, *g__norank_f__Lachnospiraceae*, *g__Eubacterium_xylanophilum_group* and *g__Roseburia* (Figure 3F), and the differential gut microbiota taxa enriched in the uridine group included *g__norank_f__Muribaculaceae*, *g__Lachnospiraceae_NK4A136_group*, *g__unclassified_f__Lachnospiraceae*, *g__norank_f__Lachnospiraceae*, *g__Lachnospiraceae_UCG-006* and *g__Eubacterium_xylanophilum_group* (Figure 3G).

### 3.4. Correlation Analysis of the Gut Microbiota Taxa

**Figure 4:**
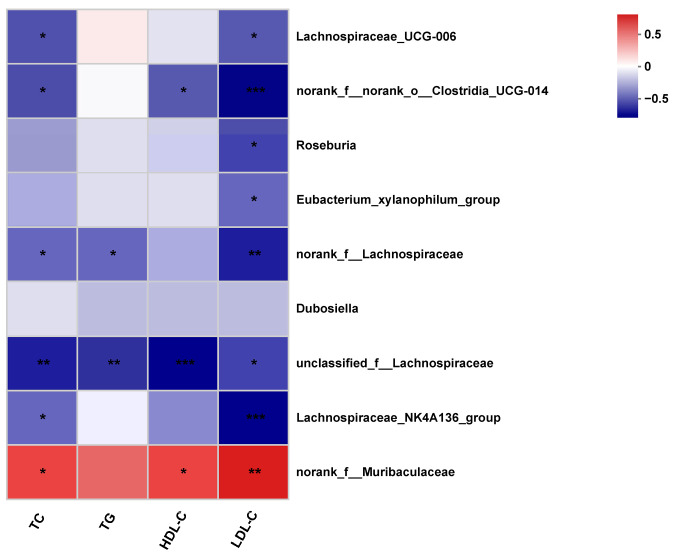
Spearman’s correlation analysis between serum TC, TG, HDL-C, LDL-C and gut microbiota taxa in the control, model, cytidine and uridine groups. * *p* < 0.05, ** *p* < 0.01, *** *p* < 0.001.

The correlation between serum TC, TG, HDL-C, LDL-C and gut microbiota taxa in the control, model, cytidine and uridine groups were analyzed according to the Spearman correlation algorithm. As revealed in Figure 4, *norank_f__ Muribaculateae* showed a significant positive correlation with all serum lipid indicators, while other gut microbiota taxa (except for *Dubosiella*) showed a significant negative correlation with serum lipid indicators.

## 4. Discussion

Ob/ob mice possess a mutation in the leptin gene, and as a result, ob/ob mice are hyperphagic, obese, and hyperglycemic and display dyslipidemia [15]. Thus, ob/ob mice were applied in this research to evaluate the effect of cytidine on metabolic disorders, especially dyslipidemia. As we can see, compared with C57BL/6J mice, the AUC values of OGTT and the serum lipid levels in ob/ob mice were significantly increased, indicating that a severe energy metabolism imbalance occurred. Additionally, it was reported that the treatment duration of uridine is closely related to lipid accumulation. Chronic uridine feeding (16 weeks) may induce severe lipid accumulation in the liver [16]. Thus, in this study, cytidine and uridine were dissolved in drinking water (0.4 mg/mL), and the treatment duration was 5 weeks. After cytidine or uridine treatment, the AUC values of OGTT in ob/ob mice showed a certain decreasing trend, but when compared with those in the model group, there was no significant difference. The levels of serum TC, HDL-C and LDL-C were significantly improved, and the hepatic steatosis was alleviated after cytidine and uridine supplementation. In summary, our results indicate that cytidine and uridine supplementation could alleviate certain aspects of dyslipidemia in ob/ob mice.

Microbiota characteristics of ob/ob mice have been extensively investigated. The ratio of *Firmicutes*/*Bacteroidetes* was closely correlated with obesity [17]. Compared with normal C57BL/J mice, the ratio of *Bacteroidetes*/*Firmicutes* in ob/ob mice is usually evidently higher, indicating that ob/ob mice suffer from metabolic disorders [18]. In our study, the ratio of *Bacteroidetes*/*Firmicutes* in ob/ob mice was 0.73, which was higher than that of C57BL/6J mice. This is consistent with the literature reported [18]. The gut microbiota has been reported to be highly associated with dyslipidemia due to its vital role in regulating host lipid metabolism [2]. Various studies were conducted, and it was revealed that the effect of the gut microbiota on dyslipidemia was mediated via microbiota-related metabolites such as short-chain fatty acids, bile acids and lipopolysaccharide [2]. In patients or experimental animals with hyperlipidemia, the abundance of short-chain fatty acids-producing microbiota and bile acids-producing microbiota was significantly decreased, while the abundance of LPS-producing microbiota was increased [19,20,21]. In this study, the abundance of *Lachnospiraceae_NK4A136_group*, *unclassified_f__Lachnospiraceae*, *Dubosiella*, *norank_f__Lachnospiraceae*, *Eubacterium_xylanophilum_group*, *Roseburia*, *norank_f__norank_o__Clostridia_UCG-014* and *Lachnospiraceae_UCG-006* were significantly decreased, and the abundance of *norank_f__Muribaculaceae* was significantly increased in ob/ob mice. Regarding *Lachnospiraceae*, *Dubosiella*, *Eubacterium_xylanophilum* and *Roseburia*, they all produce short-chain fatty acids [22,23,24]. Regarding *norank_f__norank_o__Clostridia_UCG-014*, no clear evidence showed that it could produce short-chain fatty acids; however, *Clostridium* species were reported to be closely related to diabetics [25]. *norank_f__Muribaculaceae* was revealed to be related to bile acid metabolism [26]. Therefore, depressing the growth of short-chain fatty acids-producing microbiota may account for dyslipidemia occurring in ob/ob mice. The abundance of all the other short-chain fatty acids-producing bacteria except for *Dubosiella* was significantly increased after cytidine treatment, and all these bacteria were negatively correlated with serum lipid indicators. These results suggest that cytidine could alleviate certain aspects of dyslipidemia via modulating the gut microbiota composition in ob/ob mice, especially increasing the abundance of short-chain fatty acids-producing microbiota.

Nucleotides have a role in umami taste perception, and C57BL/6J mice have higher preferences for umami-tasting solutions [27]. In the present study, uridine and cytidine were dissolved in drinking water, and this might be the reason that an increase in water consumption was observed after uridine and cytidine treatment. In recent years, the effect of drinking water on gut microbiota has been extensively investigated. It has been reported that both drinking water pH and water quality could affect the gut microbiota composition [28,29,30]. Nevertheless, the study conducted by Vanhaecke’s group suggested no significant association between gut microbiota composition and drinking water consumption [28]. Therefore, even though the water consumption increased after uridine and cytidine treatment, the changes in gut microbiota composition observed in this study are credited with uridine and cytidine treatment rather than the increase in water consumption.

It was reported that uridine treatment could ameliorate hepatic lipid accumulation in mice by modulating the gut microbiota composition [10]. Gut microbiome analysis revealed that uridine treatment could promote the growth of butyrate-producing microbiota in high-fat-diet mice, including *Odoribacter*, *Ruminococcaceae*, *Intestinimonas*, *Ruminiclostridium*, and *Lachnospiraceae* [10]. In this study, uridine was applied as a positive control, and the bacteria that changed in ob/ob mice after uridine treatment were *g__norank_f__Muribaculaceae*, *g__Lachnospiraceae_NK4A136_group*, *g__unclassified_f__Lachnospiraceae*, *g__norank_f__Lachnospiraceae*, *g__Lachnospiraceae_UCG-006* and *g__Eubacterium_xylanophilum_group*. When comparing our results with previously reported results in both ob/ob mice and high-fat-diet mice, uridine treatment could significantly promote the growth of *Lachnospiraceae*. However, the changed gut microbiota taxa after uridine treatment obtained from ob/ob mice and high-fat diet mice were different. This may be caused by the fact that the gut microbiota composition and structure of ob/ob mice and high-fat-diet mice were different [31]. 

While this study has demonstrated that cytidine supplementation could be a potential therapeutic approach for dyslipidemia, there were limitations in this study. First, the concentrations of short-chain fatty acids were not determined, and the lipid profiles in liver tissue were not fully investigated. Second, only one dose of treatment was evaluated in this study. Third, OTUs were used in 16S rRNA amplicon data analysis instead of the more modern method of ASVs (amplicon sequence variants), which could be beneficial as a comparative assessment of the data set. Fourth, additional experiments, such as fecal microbiota transplantation, are still needed to fully elucidate the relationship between the improvement in lipid metabolism and the change in gut microbiota. In summary, the present study suggested that cytidine supplementation could be a potential therapeutic approach for dyslipidemia. However, future studies to validate the efficacy and better understand the mechanism are still needed.

## 5. Conclusions

In summary, our results reveal that cytidine treatment could reduce serum lipid levels and alleviate hepatic steatosis in ob/ob mice. Modulating the composition of gut microbiota, especially promoting the growth of short-chain fatty acids-producing bacteria, might at least partially account for the activity of cytidine. Thus, cytidine supplementation could be a potential therapeutic approach for dyslipidemia.

## Data Availability

Not applicable.
